# Investigating the Ability of *Edwardsiella ictaluri* and *Flavobacterium covae* to Persist within Commercial Catfish Pond Sediments under Laboratory Conditions

**DOI:** 10.3390/pathogens12070871

**Published:** 2023-06-25

**Authors:** James T. Tuttle, Timothy J. Bruce, Ian A. E. Butts, Luke A. Roy, Hisham A. Abdelrahman, Benjamin H. Beck, Anita M. Kelly

**Affiliations:** 1 Alabama Fish Farming Center, Greensboro, AL 36744, USA; jtt0039@auburn.edu (J.T.T.);; 2 School of Fisheries, Aquaculture, and Aquatic Sciences, Auburn University, Auburn, AL 36849, USA; tjb0089@auburn.edu (T.J.B.); iab0007@auburn.edu (I.A.E.B.); 3 Department of Veterinary Hygiene and Management, Faculty of Veterinary Medicine, Cairo University, Giza 12211, Egypt; 4 Aquatic Animal Health Research Unit, US Department of Agriculture, Agricultural Research Service, Auburn, AL 36832, USA

**Keywords:** bacterial diseases, catfish, commercial aquaculture, soil microbiology, environmental adaptations

## Abstract

Two prevalent bacterial diseases in catfish aquaculture are enteric septicemia of catfish and columnaris disease caused by *Edwardsiella ictaluri* and *Flavobacterium covae*, respectively. Chronic and recurring outbreaks of these bacterial pathogens result in significant economic losses for producers annually. Determining if these pathogens can persist within sediments of commercial ponds is paramount. Experimental persistence trials (PT) were conducted to evaluate the persistence of *E. ictaluri* and *F. covae* in pond sediments. Twelve test chambers containing 120 g of sterilized sediment from four commercial catfish ponds were inoculated with either *E. ictaluri* (S97-773) or *F. covae* (ALG-00-530) and filled with 8 L of disinfected water. At 1, 2, 4-, 6-, 8-, and 15-days post-inoculation, 1 g of sediment was removed, and colony-forming units (CFU) were enumerated on selective media using 6 × 6 drop plate methods. *E. ictaluri* population peaked on Day 3 at 6.4 ± 0.5 log_10_ CFU g^−1^. Correlation analysis revealed no correlation between the sediment physicochemical parameters and *E. ictaluri* log_10_ CFU g^−1^. However, no viable *F. covae* colonies were recovered after two PT attempts. Future studies to improve understanding of *E. ictaluri* pathogenesis and persistence, and potential *F. covae* persistence in pond bottom sediments are needed.

## 1. Introduction

The commercial catfish industry is one of the largest aquaculture industries in the United States and has consistently led all aquatic species in sales from 1988 to 2018 [1]. The professionals responsible for channel catfish (*Ictalurus punctatus*) and hybrid catfish [♀ channel catfish (*I. punctatus*) × ♂ blue catfish (*I. furcatus*)] production have experienced exceptional progress in the industry but have also had to deal with numerous and recurring challenges. One of the most costly and consistent issues that commercial catfish producers face are bacterial diseases [2], namely enteric septicemia of catfish (ESC) caused by *Edwardsiella ictaluri* [3] and columnaris disease (CD) caused by *Flavobacterium covae* [4].

### 1.1. Edwardsiella ictaluri

*Edwardsiella ictaluri* is a Gram-negative, facultative, rod-shaped, weakly motile, peritrichous bacterium [2,5,6] and has been one of the causative agents for ESC outbreaks in the commercial catfish industry for nearly 50 years [7,8,9]. In 2002, instances of light (<90.7 kg), medium (90.7–907 kg), or severe (>907 kg) *E. ictaluri* outbreaks in commercial catfish species were reported to be present on 50.5%, 39.5%, and 10.0% of United States farms, respectively [10]. In east Mississippi, Peterman and Posadas [11] reported that 1.2 million catfish and 0.7 million USD were lost due to *E. ictaluri* outbreaks during the 2016 production season alone. Abdelrahman et al. [2] reported that western Alabama catfish operations have lost 3.6 million USD in foregone sales from 2015−2021 due to *Edwardsiella* spp. Infections. One reason losses due to *E. ictaluri* are not as devastating as other bacterial infections such as motile *Aeromonas* septicemia (MAS) and CD [2] could be because fingerlings more often than market-size channel fish succumb to ESC [12]. Fish that have been exposed to and recovered from an *Edwarsiella* spp. infection will have a greater immunological response and become more resistant to latent or recurrent infections [13]. The development and implementation of a live attenuated oral vaccine in 2015 [14] has dramatically reduced losses of channel and hybrid catfish fingerlings in production settings [15,16]. It has been reported that blue catfish and genetically selective strains of channel catfish can exhibit resistance to ESC infections [17,18,19]. Hybrid catfish are moderately susceptible to ESC [13,19] but are more susceptible than channel catfish when *Edwardsiella piscicida* is the causative agent [15,20]. Although notable progress has been made in managing and mitigating losses due to ESC, the disease remains to be an annual issue for catfish producers in west Alabama.

Chronic or recurring ESC outbreaks in commercial catfish ponds are commonplace [9,21,22,23] and can occur due to numerous factors. For example, the pathogenesis of *E. ictaluri* is distinct compared to other warm-water bacterial infections because it is considered an intracellular pathogen and can replicate within channel catfish macrophages [24]. Pathogenesis typically occurs horizontally when an uninfected fish cannibalizes an infected fish, as it has been known to survive the head kidney and forebrain of channel catfish [24,25,26,27]. Mqolomba and Plumb [25] reported that the head kidney, brain, blood, liver, trunk kidney, spleen, gonad, gall bladder, and muscle of fish still contained >10^4^ bacterial cells g^−1^ 65 days post-exposure to *E. ictaluri.* Surviving fish can remain carriers for *E. ictaluri* even after antibiotic treatment [28].

Another explanation for these recurring infections could be the ability of *E. ictaluri* to persist within commercial catfish ponds. In addition to *E. ictaluri*, bacterial species *E. tarda*, *F. columnare*, *Streptococcus iniae*, and *Yersinia ruckeri*, and many strains of *A. hydrophila* have been found in aquaculture pond waters and soils [29,30,31,32]. Genetic research has revealed adaptations that would allow the bacterium to survive in stressful environments. Biofilm formation by *E. ictaluri* has been reported on multiple substrates commonly found in aquaculture operations [31]. The genome of this pathogen contains sequences for six different heat shock proteins and 13 universal stress proteins that can be upregulated when exposed to oxidative stress, thermal stress, acid stress, and catfish serum stress [33]. The TonB energy transducing system and TonB-dependent transporters within *E. ictaluri* allow the pathogen to compete for and actively transport essential scarce nutrients [22]. Due to the pathogen’s ability to infect diverse fish species, *E. ictaluri* has been reported to express a high level of biochemical heterogeneity, mainly resulting in differing activities from ornithine decarboxylase, cytochrome oxidase, H_2_S production, and production of gas and acid from glucose metabolism [34,35,36]. Plumb and Quinlan [37] reported direct evidence of *E. ictaluri* surviving within the pond water for a short period and within the mud of commercial catfish pond bottom for several days. While numerous professional and academic contemporaries have widely accepted this, it remains unclear how long *E. ictaluri* would persist in a production environment more analogous to a commercial catfish pond.

### 1.2. Flavobacterium covae

Historically, the causative agent of CD in catfish aquaculture has been turbulent. In 1917, the bacterial pathogen was first named *Bacillus columnaris* due to its tendency to form haystack-like masses when sourced from external catfish lesions [38]. After successful culture conditions were determined, the bacterium was renamed *Chondrococcus columnaris* [39]. The pathogen was reclassified again in 1945 as *Cytophaga columnaris* [40], then *Flexibacter columnaris* [41], then *Flavobacterium columnare* in 1996 [42]. Recent studies have revealed genetic heterogeneity of *F. columnare* isolates worldwide [43,44], which warranted further differentiation into four distinct genomovars [45] and finally, four different species [4]. Today, and throughout this study, the primary bacterial pathogen responsible for CD outbreaks in channel and hybrid catfish aquaculture is *F. covae* [4,45].

Explanatorily, *F. covae* is a Gram-negative, aerobic, long filamentous rod-shaped, gliding, non-halophilic, yellow-pigmented, opportunistic pathogenic bacterium [4,46,47,48,49]. Outbreaks of CD in commercial catfish species can occur via direct fish-to-fish transmission when a carrier sheds the bacterium or through the water column [50]. Pathogenesis of CD occurs during periods of high fish stress when temperatures and organic loads in ponds are high, fish are overstocked, and exposed to excessive handling [51]. While the specifics of pathogenesis are not fully understood, generally, the pathogenic bacterium first colonizes the host via attraction, adhesion, and aggregation mechanisms. This is followed by the production of endotoxins, exotoxins, and bacteriocins, which eventually lead to the pathogen overwhelming the host fish’s immune system and causing the disease [52]. In addition, the mucus that naturally covers the gills and skin of catfish causes a more robust chemotactic response in *F. covae* (formerly *F. columnare* genomovar II) compared to *F. columnare* (formerly *F. columnare* genomovar I), indicating a potential relationship involving adhesion [53].

Economically, the CD has caused severe losses to the commercial catfish industry since 1922 [54]. It has been reported to be the second-highest disease observed on catfish farms in the United States [29]. Losses are estimated to be 30 million USD annually [55]. While average mortality due to CD is between 50–60%, ponds containing channel catfish fingerlings can experience up to 90% mortality [54]. In west Alabama, the highest number of fish losses were due to CD outbreaks, which equaled an estimated 14.6 million USD in foregone sales from 2015 to 2021 [2]. This discrepancy in economic loss and fish number loss is likely because fingerlings and young fish are the most susceptible to CD [56]. In similarity to *E. ictaluri* and virulent *A. hydrophila* (vAh) diseases, CD outbreaks and infections caused by *F. covae* can be chronic and recurring [48,50,51,52,56,57]. Additionally, *F. covae* has several adaptations for the bacterium to survive and potentially persist in harsh environments.

Historically, *F. covae* growth has been most successful by using low-nutrient media [39,58]. Concerning growth and prevalence, CD can be influenced by increasing temperature, organic matter, and nitrite concentration in the water [52]. Like other aquatic pathogens, *F. covae* can form biofilms in aquaculture systems, with factors such as calcium concentration, temperature, hardness, salinity, and the presence of certain carbohydrates can impact the formation of biofilm and growth [4,31,46]. Cai et al. [46] reported that the optimal conditions for *F. covae* biofilm formation are at 28 °C, 360 ppm hardness, 5 ppt salinity, and when mannose is present. Shoemaker and LaFrentz [59] have reported the capability of *F. covae* to utilize fish mucus as a nutrient source, which may alter virulence and protein expression. Some *Flavobacterium* spp. can grow at temperatures as high as 45 °C, while most are considered psychrophilic or psychrotolerant [60]. *Flavobacterium* spp. have been found in numerous environments, including bodies of freshwater and seawater, sediments, soils, glaciers, ice, and freshwater shrimp and catfish ponds [60,61,62]. Adaptations for dealing with environmental stressors such as peroxide resistance, iron metabolism, heat shock proteins, and multiple stress response mechanisms have been found within *F. columnare* and *F. covae* genomes [44,63]. The bacterium can also cope with oxidative stress and prolonged starvation, and bacterial cells can be revived following starvation while expressing less virulence [64,65].

With the ability of the opportunistic pathogens *E. ictaluri* and *F. covae* to handle harsh environments, it is plausible that they may potentially be able to persist within commercial catfish ponds over extended periods. Sediments accumulate most rapidly in the first years of pond use and, on average, can accumulate as much as 40 cm of sediment over 15 years [66]. Sediments consist of inorganic and organic matter originating from biological sources, primarily phytoplankton, catfish wastes, and uneaten feed [66,67]. Because sediment and organic materials continue to accumulate on the pond bottoms, and the drastic changes within the pond during a production season allow many opportunities for *E. ictaluri* and *F. covae* to infect stressed fish [30] and, more importantly, provide a viable environment for the pathogens to persist. The primary focus of this study was to determine if *E. ictaluri* and *F. covae* can persist within submerged pond sediments while simultaneously observing how their populations change over time. Additionally, physiochemical components of the sediments were examined to determine if they correlated with observed population trends. We hypothesized that both *E. ictaluri* and *F. covae* would successfully propagate within this environment and that differences in population growth would occur between different sediment types.

## 2. Materials and Methods

### 2.1. Previous Study

Persistence trials (PTs) using isolates of *E. ictaluri* and *F. covae* were subjected to the same experimental conditions described [68]. The PTs’ sediment samples, water, bacterial inoculum, and aquaria systems were prepared using the methods described below.

### 2.2. Experimental Design and System Preparation

All methods utilized for sediment sample collection, sediment, water disinfection techniques, and PT system preparation were the same as those described by Tuttle et al. [68]. In addition, the systems were in temperature-controlled lab spaces set to a targeted 27.5 and 27.0 °C for the *E. ictaluri* PT (EI*_PT_*) and *F. covae* PT (FC*_PT_*), respectively.

### 2.3. Bacterial Culture and Trial Preparation

The wild-type *E. ictaluri* isolate S97-773 (recovered from diseased channel catfish at the Thad Cochran National Warmwater Aquaculture Center in Stoneville, Mississippi; accession number: JX867005) was utilized for this study [14,69]. Cryopreserved S97-773 stocks were revived on brain-heart infusion (BHI) agar and incubated for 48 h at 28 °C. Next, a pure *E. ictaluri* colony was placed in 1 L of BHI broth and incubated at 28 °C and 115 revolutions per minute (RPM) for approximately 48 h. The broth culture was centrifuged at 4000× *g* for 10 min in a 5810 R benchtop centrifuge (Eppendorf North America Inc., Enfield, CT, USA), washed in cold 1X phosphate-buffered saline solution (PBS) with an adjusted pH of 7.4. Bacterial cells were resuspended and adjusted to an optical density of 0.200 ± 0.005 at 550 nm using an Eppendorf Biospectrometer^®^ Basic (Eppendorf North America Inc.), resulting in an average inoculum concentration of 8.33 × 10^7^ colony forming units (CFU) mL^−1^.

Preparation of the *F. covae* inoculum, using isolate ALG-00-530 (recovered from a diseased channel catfish at the Alabama Fish Farming Center in Greensboro, Alabama; accession number: MG516971), followed a similar procedure [70,71]. However, the culture media was modified by Shieh [58] containing the antibiotic tobramycin at a concentration of 1 mg L^−1^ of media (MST) resulting in a more selective media [72]. The *F. covae* was passed over the selective MST agar five times to ensure the bacterium had grown accustomed to the antibiotic. After the fifth pass, a pure colony of *F. covae* was placed into 1 L of Modified Shieh broth and incubated for 24 h at 28 °C and 115 RPM. Once the broth culture had grown, the bacterial cells were spun down, as mentioned previously, and instead washed with a 0.1X PBS solution with an adjusted pH of 7.0. Bacterial cells were resuspended and adjusted to an optical density of 0.200 ± 0.005 at 550 nm using a DR3900 visible spectrophotometer (Hach Company, Loveland, CO, USA) to determine bacterial concentration [73]. The final *F. covae* inoculum concentration in PBS was 1.78 × 10^7^ CFU mL^−1^.

A randomized block design was used for each PT to assign the four sediment types to the 12 total chambers. In each chamber, 20 mL of either *E. ictaluri* or *F. covae* optically adjusted bacterial inoculum was added to 200 g of sterilized sediment and 500 mL of disinfected dechlorinated city water. The sediment mixture was vigorously stirred with a sterile stainless-steel spatula for 1 min durations every 5 min over 1 h. After the mixing period, water volume within each chamber was increased to a total of 8 L. To simulate production pond aeration, a 3.5 cm × 1 cm × 1 cm cuboid Pawfly air stone at a fixed location within each chamber would expel air supplied via a Whitewater Silent Air Pump™ v201 (Pentair Aquatic Eco-Systems™, Apopka, FL, USA) for 12 h beginning at 18:00 h and stopping at 06:00 h the following day.

### 2.4. Sampling and Bacterial Enumeration

Sediment in each chamber was collected and bacterial populations were evaluated, with sampling times as follows: 24 h post-inoculation (designated as Day 0), 48 h post (Day 1), four days post (Day 3), six days post (Day 5) and eight days post (Day 7), then every seven days following the fifth sampling. Cai et al. [32] described the methodology used to extract sediment and enumerate live colonies of S97-773 and ALG-00-530 for their respective trials. Approximately 1 g of sediment was collected from each chamber using a sterile 10-mL serological pipette, placed in a sterile 15-mL centrifuge tube, and centrifuged for 10 min at 667× *g*. Liquid supernatant was removed and the remaining sediment pellet (~1 g) was resuspended entirely in 0.1X PBS, creating a 1:10 mixture, and vortexed until the pellet was homogenized. Next, 250 μL of homogenized sediment solution was placed into six wells of the leftmost column of a 96-well plate and serially diluted (10-fold), as Chen et al. [74] described. Four serial dilutions of six 10 μL replicates were each plated onto *E. ictaluri* Medium (EIM) as the selective media [75].

The spread plate method [76] and MST media were utilized to enumerate ALG-00-530 colonies. Two technical replicate MST agar plates were used for the four targeted 10-fold serial dilutions [58]. The EIM and MST plates were incubated at 28 °C for 24 h. The plate counts of *E. ictaluri* and *F. covae* were recorded, and the final counts of CFU g^−1^ of sediment were determined using the appropriate correction factors. On each sampling day, viable *E. ictaluri* and *F. covae* colonies were picked, and both were cryopreserved in a 50% glycerol stock at −80 °C. Additionally, a representative colony underwent genomic DNA extraction for later polymerase chain reaction (PCR) confirmation. Any bacteria not confirmed to be the isolates of interest were designated as “unknown” and labeled as such, followed by their respective chamber name, sampling day, and PT.

### 2.5. DNA Extraction and PCR Confirmation

After colony enumeration, the colonies of the bacterial species of interest were picked and confirmed via polymerase chain reaction (PCR) protocols. Genomic DNA (gDNA) from all bacterial colonies was extracted using the EZNA^â^ Bacterial DNA Kit (Omega Bio-tek Inc., Norcross, GA, USA). Finally, all concentrations and gDNA purity measurements were assessed using a NanoDrop™ One^C^ spectrophotometer (Thermo Fisher Scientific Inc., Waltham, MA, USA).

For the *E. ictaluri* colonies, a 25 μL PCR reaction was constructed using 12.5 μL of Hot-Start Taq Master Mix 2X (Amresco LLC, Solon, OH, USA), 1 μL of ESCF and ESCR primers from an initial 10 μM stock solution [77], 75 ng of template gDNA, and nuclease-free (NF) H_2_O to volume. Thermal cycling was conducted using an Eppendorf Mastercycler^®^ X50s (Eppendorf North America Inc.). After optimization, thermal cycling parameters consisted of an initial denaturation at 94 °C for 3 min followed by 35 cycles of 94 °C for 30 s, 58 °C for 30 s, and 72 °C for 1 min, with a final extension at 72 °C for 5 min. Positive and negative controls were run in a thermal cycler with test isolates. Then, 5 μL of PCR product was separated on a 2.0% agarose gel, stained with SYBR Safe DNA Stain (Edvotek^â^, Washington, DC, USA), in a 1.0X Tris-acetate-EDTA running buffer using electrophoresis. All gels were run containing a positive control (S97-773), negative control (NF H_2_O), and a 50 bp DNA Step Ladder (Promega, Madison, WI, USA). PCR products were visualized using a VWR^®^ Real-Time Electrophoresis Systems LED transilluminator (VWR International, Radnor, PA, USA).

To confirm *F. covae* colonies, 25 μL PCR reactions were constructed using 12.5 μL of Hot-Start Taq Master Mix 2X (Amresco LLC, Solon, OH, USA), 1.25 μL of FcFp and FcRp primers from an initial 10 μM stock solution [78], 75 ng of template gDNA, and NF H_2_O to volume. Optimized thermal cycling runs began with an initial denaturation of 95 °C for 5 min followed by 40 cycles of 94 °C for 30 s, 56 °C for 20 s, and 72 °C for 1 min, with a final extension at 72 °C for 10 min. Gel electrophoresis protocols were followed, as mentioned above.

Any colonies not PCR-confirmed as *E. ictaluri* or *F. covae* in their respective trials were designated as unknowns and labeled with the sampling day, chamber name, and PT. To accurately identify unknown bacterial colonies via the 16s rRNA gene, PCR products, and primers 63F and 1387R [79] were sent to Eurofins Genomics LLC (Louisville, KY, USA). After nucleotide base-pair results were trimmed and aligned in the Molecular Evolutionary Genetics Analysis (MEGA) software version 11 [80], base-pair sequences were inputted into the National Center for Biotechnology Information (NCBI) Basic Local Alignment Search Tool (BLAST) database [81].

### 2.6. Sediment, Water, and Statistical Analysis

All procedures for conducting water quality and sediment physiochemical analyses were the same as those described by Tuttle et al. [68].

A *t*-test was used to evaluate variances in sediment chemistry parameters. Changes in bacterial populations (log_10_ CFU g^−1^) over time among four sediment types collected from two farms were determined using a two-way repeated measures analysis of variance test, with sediment type treated as a random blocking factor. Differences between overall populations within the two PTs were measured with a paired *t*-test. Significant differences were identified via a post hoc Tukey’s Studentized Range—HSD. A correlation analysis was used to link soil chemistry parameters and bacterial populations (log_10_ CFU g^−1^). Data from each physicochemical sediment variable were analyzed for normality using a Shapiro-Wilk test. If the results did not follow the normality assumption, Spearman’s rank correlation was used. To control the false discovery rate, all multiple testing *p*-values for correlation analyses alone were adjusted using the Benjamini–Hochberg procedure [82]. A *p* < 0.05 was considered to be statistically significant.

Additionally, bacterial persistence curves (BPC) were created for each farm and each overall PT by fitting a smoothing spline (SS) model to the *E. ictaluri* and *F. covae* populations (log_10_ CFU g^−1^; y-axis) at sampling days (x-axis) as previously described by Hussain et al. [83]. The smoothing parameter (λ) was selected based on the restricted maximum likelihood (REML) method [84] to balance both function smoothness and lack of fit. The fitted SS models were used to predict *E. ictaluri* and *F. covae* populations using an x-axis scale from 0–14 d with an interval of 0.001. For each BPC, 95% confidence intervals (95% CI) of predicted bacterial population curves were created via bootstrapping [85] using the boot package, version 1.3-28; [86]. Data were resampled with replacement 1000 times, with the SS model re-fitted to both the EI*_PT_* and FC*_PT_* population data. The 95% CIs were determined from the 2.5 and 97.5th percentiles. We considered the BPC descriptors to differ significantly between farms if their 95% CIs did not overlap. The G*Power 3.1.9.4 software was used for sample size calculations [87]. BPC analyses were conducted using R software, version 4.1.1 [88]. All other statistical analyses were performed with SAS^®^ version 9.4 [89]. All figures were plotted using SigmaPlot version 14.5 (Systat Software Inc., San Jose, CA, USA). All data were presented as the mean ± standard error of the mean (SE).

## 3. Results

### 3.1. Edwardsiella ictaluri Persistence Trail

**Figure 1:**
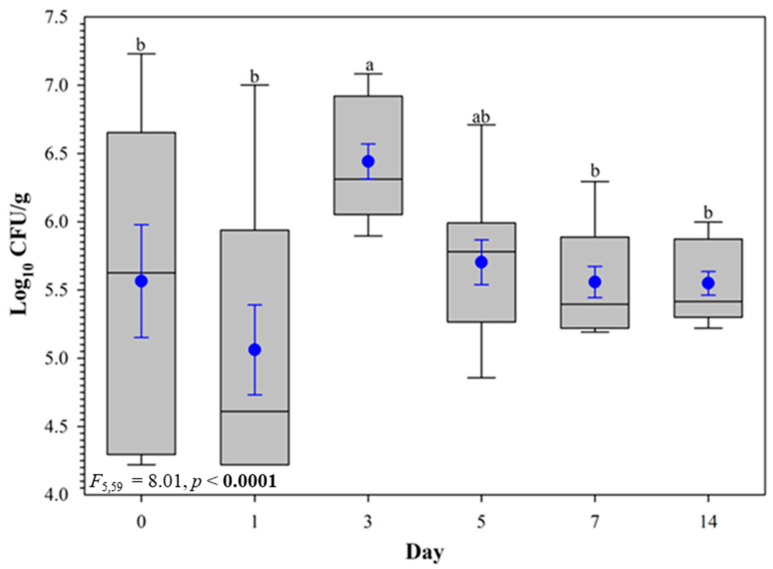
Persistence of *Edwardsiella ictaluri* S97-773 population (log_10_ CFU g^−1^) in sediment samples collected from 12 study chambers (2 farms × 2 ponds/farm × 3 replicate tanks per pond). Within each box plot, the horizontal line indicates the median, symbols indicate the mean and error bars around the symbol represent the standard error of the mean. Box plots with different lowercase letters are significantly different at *p* < 0.05.

The temperature during this persistence trial remained at 27.5 ± 0.3 °C throughout the 14-day trial. Colonies of *E. ictaluri* began appearing on the selective EIM media on Day 0 (24 h post-inoculation). However, some *E. ictaluri* growth across different replicates experienced a lag period and did not begin appearing on the EIM agar until Day 1 (48 h post-inoculation). All test chambers were inoculated and mixed within the same period of 60 min, and there was congruent growth in all sediment types but not all replicates. Despite this, populations of *E. ictaluri* initially experienced a steady increase, followed by a moderate decline and plateauing pattern over 14 days (Figure 1).

#### 3.1.1. Sampling and Bacterial Enumeration

**Figure 2:**
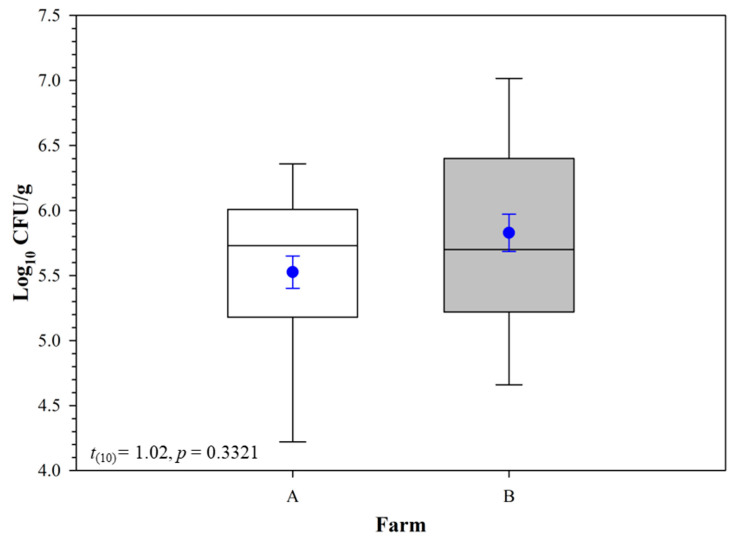
Comparison of *Edwardsiella ictaluri* population (log_10_ CFU g^−1^) in sediment samples collected from two farms (2 ponds/farm × 3 replicate tanks per pond). Within each box plot, the horizontal line indicates the median, symbols indicate the mean and error bars around the symbol represent the standard error of the mean.

**Figure 3:**
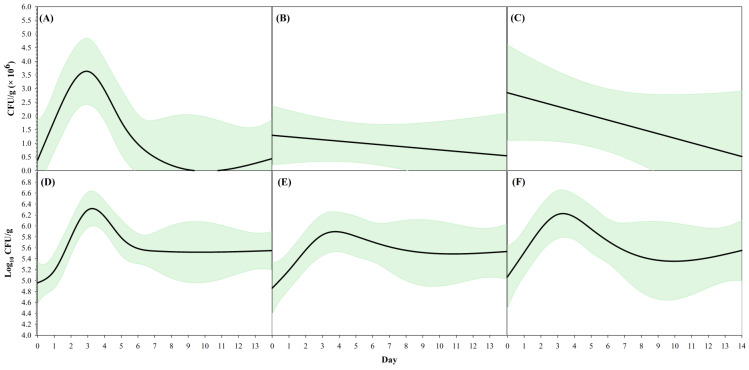
Relationship between *Edwardsiella ictaluri* population in sediment (CFU g^−1^: (**A**–**C**); log_10_ CFU g^−1^: (**D**–**F**)) and time (Days) using a smoothing spline (SS) model and 95% confidence intervals (green shadow). (**A**,**D**) represent all samples; (**B**,**E**) represent farm A; (**C**,**F**) represent farm B. Estimates of SS model descriptors are summarized in Table 1.

Across all 12 test chambers, population numbers on Day 3 were significantly higher than those on all other days (*t*_60_ = 3.55, *p* = 0.0011), except for Day 5 (*t*_60_ = 2.93, *p* = 0.0516). On Days 5, 7, and 14, the *E. ictaluri* total population was not different according to the pairwise comparisons among those respective sampling days (*p* > 0.05; Figure 1). When comparing the sediment types from the two farms, the overall population of *E. ictaluri* in farm B sediment was similar to the total population of *E. ictaluri* in farm A sediments (Figure 2). On the smoothing splines encompassed by 95% CIs, containing the raw values of CFU g^−1^ and log_10_ transformed CFU g^−1^ values (Figure 3), there are no differences in any population peak, breadth, or range values between farm A and farm B sediments, or overall sediment counts (Table 1).

#### 3.1.2. Bacterial Isolate Genetic Confirmation

**Figure 4:**
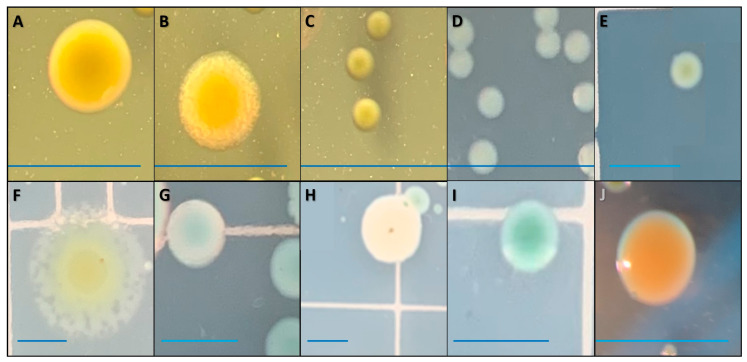
Unique bacterial colonies were visually identified on selective EIM during the EI*_PT_*. All colonies expressing different sizes, morphologies, and colors were accounted for. All blue lines next to each distinct colony represent 1000 μm. Sampling days of first appearance and identities of colony types A (**A**), B (**B**), C (**C**), D (**D**), E (**E**), F (**F**), G (**G**), H (**H**), I (**I**), and J (**J**) are listed in Table 2.

Unknown bacterial colonies first appeared in the sediment sourced from farm B on Day 1 (48 h post-inoculation) and were present in all sediment types by Day 3. The morphology and phenotypic expression of unknown bacterial colonies were more varied and diverse (Figure 4). The first colony that appeared in the selective EIM (designated as colony type A) was confirmed to be S97-773 via PCR and 16s rRNA sequencing procedures. Colony type A was present on all sampling days throughout the trial (Figure 4). On sampling Day 3, other colonies appeared to have the same color and shape but were small punctiform and pulvinate (colony type C) or intermediate-sized (colony type E). Some colonies appeared to have nearly the same morphology as ones designated as colonies A and C but began to exhibit a translucent and erose margin at both large (colony type B) and smaller sizes (colony type D). On sampling Day 7, there were large colonies that expressed white/opaque (colony type G), dark green (colony type I), and yellow (colony type J) color morphologies. Finally, on sampling Day 14, colonies that exhibited a curled and seemingly dehydrated margin (colony type H) and a noticeably larger colony size with a lobate margin (colony type F) began appearing.

**Figure 5:**
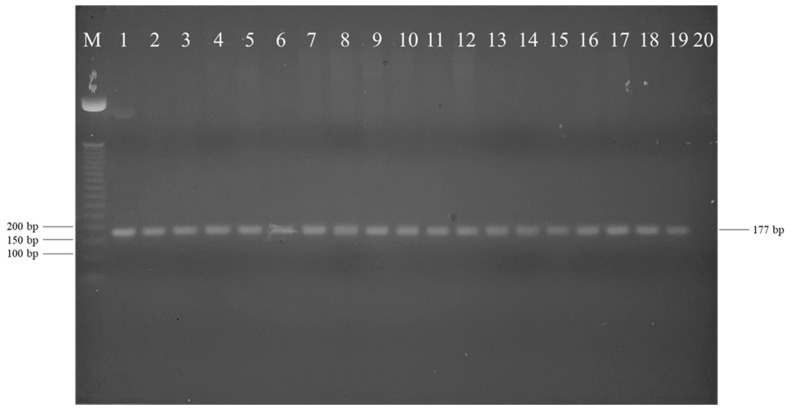
Gel electrophoresis image with visualized products of conventional polymerase chain reaction using ESCF and ESCR primers specific to *Edwardsiella ictaluri*. Bacterial isolates (arranged in the order in lanes 1–19); 1, positive control (S97-773); 2–4, colony type A (Days 0, 7, and 14); 5–7, colony type B (Days 5, 7, and 14); 8–9, colony type C (Days 1 and 7); 10–11, colony type D (Days 5 and 7); 12–13, colony type E (Days 5 and 7); 14, colony type F (Day 14); 15, colony type G (Day 7); 16, colony type H (Day 14); 17, colony type I (Day 14); 18–19, colony type J (Days 5 and 7); 20, no template, negative control; M = 50 bp DNA ladder.

Although colonies more phenotypically varied in this PT, PCR product bands using the ESCF and ESCR primers resulted in all isolated colonies producing the same amplicon region (177 bp) consistent with the positive control, indicating no apparent differentiation between the isolated bacterial colonies (Figure 5). A more robust confirmation was conducted, and the 16s rRNA sequencing revealed six different species not identified as *E. ictaluri* (Table 2). Additionally, it would appear that three distinct bacterial colonies that initially appeared to be different from the species of interest were identified as *E. ictaluri.*

#### 3.1.3. Sediment and Water Analysis

The water quality parameters did not noticeably fluctuate throughout the PT (Table 3). The sediments used in this PT are the same as the four sediment types used in a previous study, and all physical and chemical parameters between the two farms were not different [68]. Due to the small sample size of sediment physiochemical properties, the correlation analysis indicated no correlation between *E. ictaluri* populations over time and the sediment parameters (Table 4). Power analysis revealed the sample size required to determine the statistically significant correlations between *E. ictaluri* populations and each sediment parameter (Table 4). After the bacterial enumeration procedure was complete, all sediment samples were frozen.

### 3.2. Flavobacterium covae Persistence Trial

This PT was conducted on two separate occasions following all procedures described above. The water temperatures for the first and second attempts were approximately 27.2 ± 1.2 and 27.0 ± 0.4 °C, respectively. In both instances, no colonies of *F. covae* were recovered from the sediment over seven days. Incubation times were increased to 72 h to ensure that *F. covae* colonies were given ample media contact and propagation time, however, no colonies of *F. covae* propagated. Due to the lack of *F. covae* colonies being recovered in either PT, none of the statistical analyses described previously were conducted. In both FC*_PT_* attempts, viable colonies of unknown bacteria appeared on sampling Day 1 (48 h post-inoculation), displaying various unique colony morphologies. However, 16s rRNA sequencing outputs revealed that none of the colonies were *F. covae* or any *Flavobacterium* spp., revealing 12 distinct species (Table 5). Similar to the EI*_PT_*, all sediment samples were frozen after sampling.

## 4. Discussion

The results of the EI*_PT_* indicate that *E. ictaluri* can persist within the submerged sediments of commercial catfish ponds in a controlled laboratory setting. The bacterial growth curve illustrated by the data indicated that *E. ictaluri* populations began to plateau by Day 5 and did not change throughout the remainder of the trial. The highest average population across all sediments were log_10_ 6.4 CFU g^−1^. Due to no overall difference between sediments sourced from the two different farms, this suggests that sediment has no apparent influence on the growth and maintenance of pathogen populations. These findings are consistent with those Plumb and Quinlan [37] reported and displayed similarities to how vAh behaves under similar experimental conditions [68]. Like vAh, *E. ictaluri* populations experienced a growth period, followed by reaching the stationary phase, and then plateaued to remain at a consistent population. However, unlike vAh, the bacterial populations in the EI*_PT_* began plateauing by the fifth sampling day, compared to a vAh persistence trial when bacterial populations began to plateau by the fourteenth sampling day [68]. The implications of this study provide valuable insight into the ability of these pathogenic bacterial species to survive in atypical environments. These findings, as well as Tuttle et al. [68], indicate the plausibility of *E. ictaluri* and virulent *A. hydrophila* to survive within the sediments of operating commercial catfish ponds as well as potentially other aquatic environments. This study adds another layer of understanding and prompts future research to better understand bacterial pathogenesis within catfish species.

Aside from this study, there are very few publications with direct evidence regarding the ability of *Edwardsiella* spp., let alone *E. ictaluri*, to survive or persist within sediments or soils of aquatic environments. *E. ictaluri*-specific phages found in water and sediments in a river in Hiroshima Prefecture, Japan [90], have been linked to an individual forktail bullhead (*Pelteobagrus nudiceps*). Viable *E. tarda* colonies have been found in the sediments and water of aquaculture ponds, and genetic differentiation exists between isolates found exclusively in sediments versus isolates collected from other sources [91,92]. In addition, *E. tarda* found in the soils of Owerri, Nigeria, displayed potential as a species for bioremediation of crude oil [93]. Regarding sediment, the correlation analysis could not distinguish significant physical and chemical factors of the sediment that influenced the population of the bacterial pathogen. The power analysis revealed a larger sample size is necessary to determine statistical significance with high power. These sample size numbers, which were in the thousands, would be unrealistic and cost prohibitive within the scope of this study but would be worth future investigation. It is also necessary to determine which cellular mechanisms and virulence factors allow for the persistence of *E. ictaluri* in the sediments of catfish ponds.

It has been established that species of *Edwardsiella* are naturally resistant to colistin [75]; however, it is notable that the bacteria identified in this study exhibited colistin resistance consistent with previous research findings. Genera from the family Enterobacteriaceae, such as *Salmonella* spp., *Klebsiella* spp., *Aeromonas* spp., *Citrobacter* spp., *Shigella* spp., *Enterobacter* spp., and *Escherichia* spp. contain mobilized colistin resistance genes [94]. *Clostridium perfringens*, *Bacillus subtilus*, *Neisseria meningitides*, *Burkholderia* spp., *Proteus mirabilis*, *Kluyvera* spp., *Cronobacter sakazakii*, *Raoultella ornithinolytica*, and *Pseudomonas aeruginosa* have all shown some level of resistance to colistin [95,96,97,98]. The most interesting finding from this study comes from verifying the identities of diverse colonies that grew on the selective EIM.

Based on the results of the 16s sequencing, it is apparent that four bacterial colonies were expressing different pigmentation and sizes that were all determined to be *E. ictaluri*. One reason *E. ictaluri* was chosen for this study, as opposed to *E. tarda* or *E. piscicida*, is due to the reported high phenotypic and biophysical homogeneity among isolates [99,100]. During the beginning of the persistence trial, *E. ictaluri* colonies produced a green pigment on the EIM; however, some apparent *E. ictaluri* colonies began expressing a yellow pigment by the fifth sampling day. One study reported that *E. tarda* colony pigmentation was black when grown on Salmonella-Shigella agar [101]. Bacterial pigmentation is quite diverse, and all unique pigments have a specific function essential for bacterial survival and ecological success [102]. Different bacterial genera, including *Pseudomonas* spp., *Janthinobacterium* spp., *Streptomyces* spp., *Nocardia* spp., *Thermomonospora* spp., *Microbispora* spp., *Streptosporangium* spp., *Rhodococcus* spp., and *Kitasatospora* spp., have diverse pigmentation [103]; however, there have been no studies reporting one species or genus of bacteria being capable of expressing two different pigments. Johansen et al. [104] demonstrated through genetic modification of the motility, cell shape, stringent response, and tRNA modification genes of a *Flavobacterium* spp. strain Iridescent 1, which could alter the nanostructure, which resulted in multiple colors observed among the same bacterial colonies. They also suggested that the structural color of bacterial colonies may be linked to cellular functions and gene activity, which may have significant implications for natural populations of pathogenic bacteria. To date, no studies have determined the natural pigments produced by *E. ictaluri* or if there is a linkage between cellular functions and pigmentation. Whole genome sequencing of the isolates collected during the EI*_PT_* would reveal what pigments these bacteria can produce and add another level of confirmation to the identity of these bacteria.

Even though both attempts to propagate *F. covae* colonies within this experimental design were unsuccessful, these findings are intriguing. Multiple factors may have influenced the lack of *F. covae* in these laboratory persistence trials. Environmental conditions such as water hardness, high temperature, organic matter, and nitrite concentration can increase the adhesion and virulence of the bacterial pathogen [105,106,107]. The biofilm formation is most effective between 25–28 °C and can be inhibited when salinity is as low as 3 ppt and significantly reduced at salinities over 7.5 ppt [46,107]. Another factor could be due to ecological interactions and interspecific competition. Bacterial species including *Bacillus subtilis*, *Luteimonas aestuarii*, *Rhodococcus qingshengii*, *Leucobacter luti*, and *Dietzia maris* were antagonistic towards *F. covae* and *F. psychrophilum* [30,108,109]. Additionally, tannic acid can act as an effective bactericide for *F. columnare* and *E. ictaluri* [110]. It could be possible that some of the other bacteria that appeared in the FC*_PT_* and some natural compounds or ions in the sediment prevented the establishment of *F. covae*.

The culture conditions necessary for successful *F. covae* growth can be sensitive. Although previous studies have reported that the best growth of *F. covae* is on low nutrient media [58,73,111], and the bacteria are slow growing [112,113]. While Shieh media has typically allowed for fast and effective growth of *Flavobacertium* spp. [114], a recent study indicated that G media provides effective and uniform distribution of *F. covae* colonies within 24 h [113]. Other media, such as tryptone yeast extract salt media [112] and antibiotics such as polymyxin-neomycin have been utilized to create selective media [73] for successful *F. covae* growth. Media type and culture considerations for future persistence studies may yield more favorable results.

Since we know the pathogen *F. covae* and other members of the *Flavobacterium* genus have been found in aquatic environments outside of a host [50,115,116,117,118], it is plausible that *F. covae* may have the ability to persist within the environment. In addition to biofilm formation, a recent study by Abdelhamed et al. [119] revealed that *F. covae* could grow under anaerobic conditions via denitrification genes and nitrite reduction. However, at this time, we were unable to verify the ability of the pathogen *F. covae* to persist within the sediments of commercial catfish ponds under the conditions outlined in this study.

## 5. Conclusions

Understanding the mechanisms that allow these pathogenic bacteria to persist within sediments is vital for effective disease management strategies for commercial catfish producers. *E. ictaluri* has been confirmed to be able to persist within aquatic sediments based on the results of this study, however, this may have profound implications. Further gene expression analyses such as those conducted on vAh [120] may reveal that cell origins of *E. ictaluri* may result in different virulence factor expressions. Additionally, *E. ictaluri* persisting within sediments may be more susceptible to developing anti-microbial resistance [121], as has been reported in previous studies [36,122,123,124]. Conversely, since *F. covae* propagation was unsuccessful, modifications to this experimental design will be necessary for future studies. While *F. covae* can form biofilms, other environmental and experimental factors within the aquatic environment may contribute to them not being recovered in this study.

## Figures and Tables

**Table 1:** Mean, standard error (*SE*), and 95% confidence intervals (95% C.I.) for descriptors of smoothing spline models are presented in Figure 3.

Curve Descriptor		Overall		Farm A		Farm B	
		Mean ± SE	95% C.I.	Mean ± SE	95% C.I.	Mean ± SE	95% C.I.
**CFU g^−1^**	Peak CFU g^−1^ (× 10^6^)	3.63 ± 1.31	1.08–6.19	1.29 ± 1.78	−2.20–4.78	2.85 ± 1.97	−1.01–6.71
	Time (day) at peak CFU g^−1^	2.93 ± 0.90	1.17–4.69	0.00 ± 2.21	−4.32–4.32	0.00 ± 1.63	−3.19–3.19
	Time (day) at 90% of peak CFU g^−1^—lower	2.17 ± 0.72	0.76–3.57	0.00 ± 1.38	−2.70–2.70	0.00 ± 1.18	−2.32–2.32
	Time (day) at 90% of Peak CFU g^−1^—upper	3.68 ± 0.72	2.27–5.08	2.41 ± 2.42	−2.33–7.15	1.71 ± 1.5	−1.23–4.64
	Breadth at 90% of peak CFU g^−1^	1.51 ± 0.55	0.44–2.58	2.41 ± 2.36	−2.21–7.03	1.71 ± 0.96	−0.17–3.59
	Time (day) at 80% of peak CFU g^−1^—lower	1.81 ± 0.64	0.55–3.07	0.00 ± 1.16	−2.28–2.28	0.00 ± 0.98	−1.93–1.93
	Time (day) at 80% of peak CFU g^−1^—upper	4.04 ± 0.65	2.76–5.32	4.83 ± 2.71	−0.48–10.13	3.42 ± 1.39	0.70–6.14
	Breadth at 80% of peak CFU g^−1^	2.23 ± 0.87	0.53–3.93	4.83 ± 3.08	−1.22–10.87	3.42 ± 1.44	0.60–6.24
	Time (day) at 5% of peak CFU g^−1^—min	0.00 ± 0.20	−0.39–0.39	0.00 ± 0.22	−0.43–0.43	0.00 ± 0.20	−0.39–0.39
	Time (day) at 5% of peak CFU g^−1^—max	14.00 ± 0.47	13.08–14.92	14.00 ± 0.80	12.43–15.57	14.00 ± 1.77	10.52–17.48
	Range at 5% of peak CFU g^−1^	14.00 ± 0.51	13.00–15.00	14.00 ± 0.83	12.37–15.63	14.00 ± 1.78	10.51–17.49
**Log_10_ CFU g^−1^**	Peak log_10_ CFU g^−1^	6.32 ± 0.19	5.95–6.68	5.89 ± 1.46	3.04–8.75	6.23 ± 0.32	5.59–6.86
	Time (day) at peak log_10_ CFU g^−1^	3.27 ± 0.68	1.93–4.61	3.77 ± 3.11	−2.32–9.87	3.31 ± 2.62	−1.81–8.44
	Time (day) at 90% of peak log_10_ CFU g^−1^—lower	1.85 ± 0.50	0.86–2.83	1.32 ± 0.97	−0.58–3.21	1.20 ± 0.78	−0.33–2.73
	Time (day) at 90% of peak log_10_ CFU g^−1^—upper	5.37 ± 3.8	−2.08–12.81	14.00 ± 3.63	6.88–21.12	6.66 ± 4.12	−1.41–14.73
	Breadth at 90% of peak log_10_ CFU g^−1^	3.52 ± 4.03	−4.37–11.42	12.68 ± 4.08	4.69–20.67	5.46 ± 4.61	−3.58–14.50
	Time (day) at 80% of peak log_10_ CFU g^−1^—lower	0.61 ± 0.65	−0.66–1.87	0.00 ± 0.92	−1.81–1.81	0.00 ± 0.55	−1.09–1.09
	Time (day) at 80% of peak log_10_ CFU g^−1^—upper	14.00 ± 0.00	14.00–14.00	14.00 ± 0.31	13.39–14.61	14.00 ± 0.89	12.26–15.74
	Breadth at 80% of peak log_10_ CFU g^−1^	13.40 ± 0.65	12.13–14.66	14.00 ± 1.00	12.04–15.96	14.00 ± 1.11	11.83–16.17

**Table 2:** Results from the NCBI BLAST database for nucleotide 16s rRNA sequences from isolates collected during EI*_PT_* and the sampling day durations the unique colonies were present. Bacterial species were determined to have the highest probability under percent maximum identity (Max Ident.), highest total score, and highest maximum query cover to show the percentage of query DNA covered.

Colony Morphology	Sampling Day(s)	Confirmation			
		Total Score ^a^	Query Cover ^b^	Max Ident. ^c^	Species ID
D	5, 7	1762	100	99.37	*Burkholderia contaminans*
F	14	639	98	97.62	Uncultured bacterium
H	14	1954	100	99.53	*Bacillus* spp.
I	14	1599	100	98.84	*Pseudomonas aeruginosa*
C	1−14	1882	94	81.48	*Clostridium hydrogeniformans*
G	7, 14	1792	100	99.59	*Stenotrophomonas pavanii*
A	0−14	1677	100	99.61	*Edwardsiella ictaluri*
B	5−14	1628	100	98.13	*Edwardsiella ictaluri*
E	5, 7	1988	100	99.27	*Edwardsiella ictaluri*
J	7, 14	1831	100	98.90	*Edwardsiella ictaluri*

^a^ Sum of alignment scores of all segments from the same subject sequence. ^b^ Percent of the query length that is included in the aligned segments. ^c^ Highest percent identity for a set of aligned segments to the same subject sequence.

**Table 3:** Water quality parameters [mean, standard error (*SE*), minimum measurement (min), and maximum measurement (max)] measured in 12 study tanks containing sediment samples collected from two farms (2 ponds per farm; 3 replicate tanks per pond) for 14 d EI*_PT_*.

Water Quality Parameter	Overall		Farm A		Farm B	
	Mean ± SE	Min–Max	Mean ± SE	Min–Max	Mean ± SE	Min–Max
Total alkalinity (ppm)	116.72 ± 3.60	87–174	109.50 ± 4.30	87–157	123.94 ± 5.37	90–174
Total hardness (ppm)	123.78 ± 5.03	67–191	105.44 ± 4.92	67–138	142.11 ± 6.36	99–191
pH	7.62 ± 0.02	7.3–7.9	7.57 ± 0.03	7.3–7.8	7.67 ± 0.03	7.4–7.9
Phosphate (ppm)	1.28 ± 0.20	0.0–4.0	1.62 ± 0.32	0.0–4.0	0.94 ± 0.21	0.0–2.8
Total ammonia nitrogen (ppm)	0.60 ± 0.11	0.0–2.2	0.32 ± 0.08	0.0–1.3	0.88 ± 0.18	0.1–2.2
Nitrite (ppm)	0.11 ± 0.04	0.0–1.0	0.16 ± 0.08	0.0–1.0	0.06 ± 0.02	0.0–0.3
Nitrate (ppm)	0.15 ± 0.06	0.0–1.0	0.0 ± 0.0	0.0–0.0	0.31 ± 0.11	0.0–1.0

**Table 4:** Results from correlation analysis tests between log_10_ CFU g^−1^ of *Edwardsiella ictaluri* and sediment physicochemical variables. Based on bivariate normality testing, Spearman’s rank correlation (coefficient = *ρ*) was used. All raw *p*-values were adjusted using the Benjamini-Hochberg procedure to control the false discovery rate (FDR). Significant results at *p* < 0.05. The sample size (*n*) is required to reveal statistically significant correlations.

Variable	*ρ*	*p*-Value		*n*/Farm
		Raw	FDR	
Alkalinity (% CaCO_3_ Equivalence)	0.05	0.6740	0.2800	2728
Aluminum (ppm)	−0.04	0.7617	0.9694	5249
Calcium (ppm)	−0.04	0.7317	0.9694	5249
CEC (meq 100 g^−1^)	−0.04	0.7414	0.9694	5249
Copper (ppm)	0.08	0.5447	0.9694	1224
Iron (ppm)	0.02	0.8857	0.9694	19,260
Magnesium (ppm)	0.09	0.4627	0.9694	896
Manganese (ppm)	<0.01	0.9730	0.9694	422,523
Organic Matter (%)	0.03	0.8450	0.9694	12,627
pH	−0.02	0.9080	0.9694	36,124
Phosphorus (ppm)	0.07	0.6006	0.9694	1763
Potassium (ppm)	0.08	0.5315	0.9730	1234
Sodium (ppm)	−0.13	0.3089	0.9730	467
Zinc (ppm)	0.10	0.4373	0.9730	801

**Table 5:** Results from the NCBI Blast database for nucleotide 16s rRNA sequences from isolates collected during FC*_PT_* and the sampling day durations the unique colonies were present. Bacterial species were determined to have the highest probability under percent max identity, highest total score, and highest max query cover to show the percentage of query DNA covered.

Colony Morphology	Sampling Day(s) (FPT Attempt)	Confirmation			Species ID
		Total Score ^a^	Query Cover ^b^	Max Ident. ^c^	
A	3–7 (1, 2)	1783	100	99.90	*Brevibacterium sediminis*
B	1–5 (1, 2)	1670	100	100.00	*Micrococcus luteus*
C	1–7 (1, 2)	2021	100	99.91	*Micrococcus* sp.
D	3–7 (1, 2)	1599	100	98.74	*Sphingobium yanoikuyae*
E	3, 5 (1)	1916	99	97.10	*Acinetobacter schindleri*
F	7 (2)	1286	99	89.88	Uncultured Bacterium
G	3–7 (1)	1982	100	98.57	*Massilia neuiana*
H	3–7 (1, 2)	1857	100	100.00	*Stutzerimonas stutzeri*
I	5, 7 (2)	1988	100	99.81	*Bacillus pseudomucoides*
J	7 (2)	1607	100	99.41	*Azospirillum brasilense*
K	7 (1,2)	1700	100	100.00	*Achromobacter marplate*
L	7 (1, 2)	1825	100	99.50	*Cytiolbacillus* sp.
M	7 (1, 2)	1858	98	95.99	*Bacillus firmus*

^a^ Sum of alignment scores of all segments from the same subject sequence. ^b^ Percent of the query length that is included in the aligned segments. ^c^ Highest percent identity for a set of aligned segments to the same subject sequence.

## Data Availability

All data from this study are available from the corresponding author upon reasonable request.
